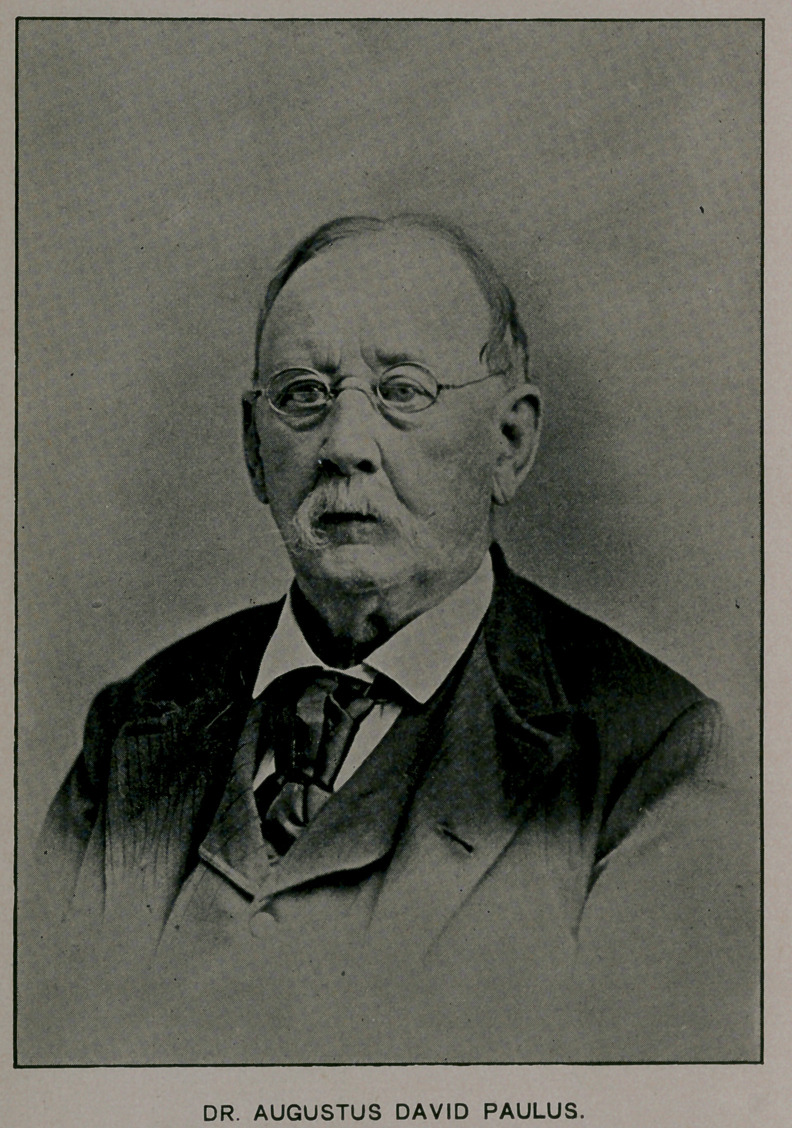# Dr. A. D. Paulus

**Published:** 1895-10

**Authors:** 


### EDITORIAL DEPARTMENT.

F. E. DANIEL, M. D., Editor.
S. E. HUDSON, M. D., Managing Editor.
A. J. SMITH. M. D., Galveston, Associate Editor.

EDITORIAL STAFF:

PROF. J. E. THOMPSON, M. D., Texas Medical College, Galveston; Surgery.
PROF. WM. KEILLER, M. D., Texas Medical College, Galveston; Obstetrics and Gynecology.
PROF. DAVID CERNA, M. D., Texas Medical College, Galveston; Therapeutics.
PROF. A. J. SMITH, M. D., Texas Medical College, Galveston; Medicine
DR. R. H. L. BIBB, Saltillo, Mexico; Foreign Correspondent.

Official organ of the West Texas Medical Association, the Houston District Medical Association, the Austin District Medical Society, the Galveston County Medical Society, and several others.

### DR. A. D. PAULUS.

Within a few short weeks since the loss of the great and good Cupples, the JOURNAL is called upon to chronicle the death of another of the pioneers and landmarks of Texas medicine. Dr. A. D. Paulus, of Flatonia, Texas, after a long and eventful life, nearly sixty years of which were spent in the practice of medicine, died at his home on the 4th of September ult., aged 78 years and 16 days.

For some years previous to his death, Dr. Paulus had been much afflicted with rheumatism, which in a great measure took him out of the active practice, but at all times he was ready with his counsel and advice to assist his brethren and minister to the needs of suffering humanity. His last illness was only of a few days duration, however, and Dr. H. A. Tutwiler, between whom and him a friendship of more than twenty-five years had existed, laid aside all professional work and all else, and remained at the bedside night and day. At 3 p. m., September 4th, he wrote Dr. Daniel, of the JOURNAL: "I am sitting at the bedside of our dear old friend Dr. Paulus. A few more minutes will end his strange, eventful career. He has only been sick three days. His sons are both here. His death will break up a friendship between him and me of over twenty-seven years. I write now that you may come, as the train from Austin will reach here at 1 p. m., and the funeral will take place shortly after."

The following data has been kindly furnished the JOURNAL jointly by Messrs. Paulus, sons of the deceased, and Dr. Tutwiler, and we are indebted to the family also for the photograph from which the accompanying cut was made:

AUGUSTUS DAVID PAULUS was the son of Dr. Jurgen Christian Paulus, of Denmark, and was born in Copenhagen, July 18, 1817. His father removed, with his family, to Kiel, Germany, shortly afterwards. Here the son grew to manhood, and having in early life entered the literary department of the University at Heidelburg, was soon able to graduate. He then studied medicine, and entering the Medical Department of the University of Kiel was graduated M. D., and received his diploma in the year 1839. He at once went to London, and spent six months in Guy's Hospital. The following year, 1840, Dr. Paulus came to America, and at once entered government service, receiving an appointment as Assistant Surgeon in the Medical Department of the United States Navy. Being assigned to duty on the United States Man of War "Missouri," he made several voyages, and was at his post when that vessel burned, in 1843. He then resigned his commission, and engaged in the practice of medicine, first at Vevay, Indiana, where he met and married his first wife, Miss Lucy Peabody, of that place. There were born of this union two children, one of whom, now Mrs. Wiebke Murphy, of Vevay, Indiana, survives him. In 1858, Dr. Paulus, having removed to Hickman, Tennessee, was again married, his second wife being Miss Mary A. Mayberry, of Centerville, Tennessee,—the mother of the two sons, Mr. J. H. Paulus, of Flatonia, and Mr. D. A. Paulus, of Hallettsville, and of the two daughters, Mrs. Mary A. Mahoney and Mrs. C. C. Harris, of Denver, Colorado, who, with her, survive him.

In 1861, Dr. Paulus removed to Texas. He located first at Gatesville, in Coryell county. This was at that time a frontier county, and as he practiced throughout several adjacent counties, he necessarily underwent many hardships and exposures; the county being at that time liable to frequent incursions by hostile Indians, it was often that he was called upon to take his life in his hand, as it were, when called on to cross the trackless prairie to visit some lone pioneer settler's cabin. It was during his residence here that the war between the States came on. Dr. Paulus accepted the position of surgeon in Major Erath's company of Texas troops, stationed on the frontier of Texas, and assisted in protecting it from the Indians. In 1867, after the war, he removed to Fayette county, settling first at High Hill, removing thence, in 1886, to Flatonia, where he resided continuously till the day of his death, practicing his beloved profession for rich and poor alike, without discrimination.

Dr. Paulus was a man of very pronounced characteristics,—strong in his likes and dislikes,—a man, however, of large sympathy, and at the sight of want or suffering his great heart went out alike to friend and foe. He was also liberal in his charity, and many of the poor of his county will mourn the loss of a benefactor. In his death, the medical profession suffers the loss of one of its ablest and most respected members, and the community a useful, public spirited citizen. The writer feels that he has sustained a personal loss, and been bereft of one of his dearest and most attached friends.

Dr. Paulus was an Odd Fellow, having attached himself to the order as early as 1844, and in 1853 became a Master Mason. He was ex-president of the West Texas District Medical Association, which body will doubtless take suitable action in commemoration of his life and services. During his entire life, he took an active part in politics, and was a staunch, life long Democrat. He did much to assist his party in organizing power in Fayette county, during the heated campaign of 1892-94. In 1869, he was elected county commissioner, the only office he would ever consent to hold. He was a close student, and read a great deal, spending his leisure time amongst his books, and for recreation cultivated many rare plants, amongst which he spent many hours, his garden of flowers at High Hill being one of the curiosities and attractions of that section, and said to be the finest collection in the State. In his friendships, he was peculiar; he either took a strong dislike to those he met, or formed for them a strong attachment. It was by his efforts that the medical profession of Fayette county was organized, he being its first president.

He was lovely in his family, a devoted father and model husband. His principal object in life, he used to say, aside from doing all the good he could to others, was to raise and educate his children and fit them for useful positions in life. In this ambition, he was fully gratified, living to see them all grown and equipped for life, and no longer dependent upon him. May his soul rest in peace.

## Figures and Tables

**Figure f1:**